# Supplementary material for: Full-length Plasmodium falciparum myosin A and essential light chain PfELC structures provide new anti-malarial targets
Source: eLife. 2020 Oct 13;9:e60581. doi: 10.7554/eLife.60581 (PMC7553781; doi:10.7554/eLife.60581)
Supplement: Supplementary file 1. — (a) Data collection and refinement statistics (molecular replacement). (b) Kinetic and motility parameters of PfMyoA mutants. (c) Dissociation of acto-PfMyoA by MgATP at 20°C. [file elife-60581-supp1.docx]

**Supplementary file 1a.** **Data collection and refinement statistics (molecular replacement)**

|  | **PfMyoA•FL-PR** | **PfMyoA•FL-PPS** | **PfMyoA•ΔNter-PR** |
| --- | --- | --- | --- |
| **Data collection** |  |  |  |
| Space group | P 2_1_ 2_1_ 2_1_ | P 2_1_ 2_1_ 2 | P 2_1_ 2_1_ 2_1_ |
| Cell dimensions |  |  |  |
| *a*, *b*, *c* (Å) | 89.67 114.69 169.46 | 168.24 287.43 78.61 | 90.08 114.43 170.70 |
| α, β, γ (°) | 90 90 90 | 90 90 90 | 90 90 90 |
| Resolution (Å) | 25.6-2.55(2.641-2.55)* | 48.4-3.99 (4.133-3.99) | 48.3-3.27 (3.387-3.27) |
| *R*_merge_ | 0.169 (1.918) | 0.4443 (2.491) | 0.3707 (2.157) |
| *I* / σ*I* | 10.57 (0.83) | 4.19 (0.64) | 9.02 (1.68) |
| CC_1/2_ | 0.998 (0.505) | 0.989 (0.319) | 0.992 (0.553) |
| Completeness (%) | 99.62 (97.94) | 99.49 (96.58) | 99.88 (99.85) |
| Redundancy | 11.2 (11.5) | 10.3 (10.1) | 12.8 (12.9) |
|  |  |  |  |
| **Refinement** |  |  |  |
| Resolution (Å) | 25.57-2.55 (2.62-2.55) | 48.4-3.99 (4.11-3.99) | 48.3-3.27 (3.39-3.27) |
| No. reflections | 641 293 (total)  57 507 (unique) | 343 030 (total)  33 243 (unique) | 358 945 (total)  27 977 |
| *R*_work_ / *R*_free_ | 0.199 / 0.248 | 0.237 / 0.273 | 0.184 / 0.232 |
| No. atoms |  |  |  |
| Protein | 8571 | 17 386 | 8490 |
| Ligand/ion | 47 | 66 | 51 |
| Water | 290 | 0 | 4 |
| *B*-factors |  |  |  |
| Protein | 79.82 | 70.84 | 92.81 |
| Ligand/ion | 83.16 | 23.55 | 78.44 |
| Water | 69.70 |  | 63.85 |
| R.m.s. deviations |  |  |  |
| Bond lengths (Å) | 0.014 | 0.014 | 0.015 |
| Bond angles (°) | 1.82 | 1.65 | 1.82 |

*Number of crystals for each structure should be noted in footnote. *Values in parentheses are for highest-resolution shell.

**Supplementary file 1b. Kinetic and motility parameters of PfMyoA mutants**

| PfMyoA  construct | In vitro motility speed  (µm/s ± SD)* | ADP release rate  (s^-1^ ± SD)* | ATPase  V_max_  (s^-1^ ± SE)* | ATPase  K_m_  (µM ± SE)* | Rate of acto-PfMyoA dissociation by MgATP  (s^-1^)^5*^ | Basal ATPase  (s^-1^)* | Ensemble force  (nM utrophin  ± SE)* |
| --- | --- | --- | --- | --- | --- | --- | --- |
| WT^1^ | 3.88 ± 0.54  (1) | 334 ± 36  (1) | 138 **±** 4  (1) | 30.3 **±** 2.3  (1) | 326 ± 9  (1) | 0.3  (1) | 1.40 ± 0.08  (1) |
| E6R | 1.91 ± 0.35  (0.49) | 157 ± 8  (0.47) | 61.8 **±** 0.9  (0.45) | 4.1 **±** 0.3  (7.39) | 857 ± 22  (2.62) | 0.5  (1.67) | 4.02 ± 0.31  (2.9) |
| S691G | 5.06 ± 0.58  (1.30) | 580 ± 31  (1.74) | 70.3 **±** 1.4  (0.51) | 15.4 **±** 1.0  (1.97) | 348 ± 7  (1.07) | 2.7  (9) | 1.99 ± 0.19  (1.4) |
| T586F | 4.04 ± 0.44  (1.04) | 323 ± 54  (0.97) | 50.1 **±** 1.4  (0.36) | 13.6 **±** 1.3  (2.23) | 471 ± 27  (1.44) | 2.1  (7) | 2.38 ± 0.18  (1.7) |
| LRA^2^ | 1.19 ± 0.18  (0.31) | 117 ± 4  (0.35) | 46.8 **±** 1.0  (0.34) | 9.1 **±** 0.6  (3.33) | 472 ± 35  (1.45) | 0.7  (2.3) | n.d. |
| AAA^3^ | 1.24 ± 0.24  (0.32) | 121 ± 2  (0.36) | 42.7 **±** 0.9  (0.31) | 12.3 **±** 0.8  (2.46) | n.d. | 0.5  (1.67) | n.d. |
| minus PfELC | 1.75 ± 0.32**^4^**  (0.45) | n.d. | n.d. | n.d. | n.d. | n.d. | 1.57 ± 0.18  (1.1) |

*Values inside parentheses are normalized relative to WT as 1.

^1^ Data from^5^

^2^ LRA: R707L/E711R/Y714A

^3^AAA: R707A/E711A/Y714A

^4^Data from^7^

^5^Temperature, 20°C. See **Supplementary file 1c** for additional values.

n.d., not determined

| **PfMyoA construct** | **V_max_**  **(s^-1^)** | **K_m_**  **(µM)** |
| --- | --- | --- |
|  |  |  |
| WT | 326 ± 9 | 240 ± 18 |
| E6R | 857 ± 52 | 351 ± 51 |
| K764E | 801 ± 54 | 257 ± 38 |
| ∆N | 616 ± 45 | 103 ± 25 |
| S19A | 592 ± 40 | 227 ± 44 |
| S691G | 348 ± 7 | 201 ± 12 |
| T586F | 471 ± 27 | 364 ± 50 |
| LRA | 472 ± 35 | 198 ± 42 |

**Supplementary file 1c. Dissociation of acto-PfMyoA by MgATP at 20°C.**

Fits to Michaelis-Menten equation ± SE of the fit.
